# Optimizing Immunization Strategies for Individuals Living with HIV: A Review of Essential Vaccines, Vaccine Coverage, and Adherence Factors

**DOI:** 10.3390/vaccines13080798

**Published:** 2025-07-28

**Authors:** Lina M. Würfel, Anja Potthoff, Adriane Skaletz-Rorowski, Sandeep Nambiar, Nessr Abu Rached

**Affiliations:** 1 Walk In Ruhr [WIR]—Centre for Sexual Health and Medicine, Große Beckstraße 12, 44787 Bochum, Germany; anja.potthoff@klinikum-bochum.de (A.P.); adriane.skaletz-rorowski@kklbo.de (A.S.-R.); sandeep.nambiar@klinikum-bochum.de (S.N.); 2 Interdisciplinary Immunological Center, Centre for Sexual Health and Medicine, Department of Dermatology, Venerology und Allergology, Ruhr University Bochum, Große Beckstraße 12, 44787 Bochum, Germany; 3 St. Josef Hospital Bochum, Department of Dermatology, Venerology und Allergology, Ruhr University Bochum, Gudrunstraße 56, 44791 Bochum, Germany; nessr.aburached@klinikum-bochum.de

**Keywords:** HIV, PLWH, vaccines, immunodeficiency, immunity, review, vaccine recommendations

## Abstract

Human immunodeficiency virus (HIV) infection remains a major challenge in global health. In recent years, vaccines have emerged as an important tool for the treatment and prevention of HIV-related complications. This review article addresses the evolving landscape of vaccines for people living with HIV (PLWH), evaluating current vaccination strategies for standard vaccines and travel vaccines in PLWH compared to the general population and offering a summary of the current recommended vaccines. It evaluates studies for vaccine effectiveness and safety and discusses methods to improve vaccination rates among PLWH. Systematic research was carried out using keywords. We address the current state of knowledge and highlight areas for future research and development.

## 1. Introduction

The human immunodeficiency virus (HIV) has remained a global health challenge for decades. HIV is a severe infectious disease characterized by a gradual decline in cellular defenses. This is evident in the heightened occurrence of opportunistic infections and specific tumors. Vaccines have emerged as an essential tool in the management and prevention of HIV-related complications.

Vaccination rates among PLWH remain suboptimal [1]. According to a study conducted in PLWH, 59% were missing one or more vaccinations [2]. Hesitance to vaccinate may be caused by limiting factors like lack of education, lack of insurance coverage, patients’ fear of suffering from adverse effects, and the tendency for other pressing health issues to overshadow vaccination discussions during healthcare visits [2,3]. Incorporating vaccination guidelines into everyday medical practices could contribute to enhancing vaccination rates among vulnerable subgroups such as PLWH [4]. Strong predictors of adherence include attending two or more annual clinic visits, which indicates that consistent engagement in care enhances the likelihood of completing vaccination [1]. To ensure efficient and uniform vaccination strategies, concise and globally applicable guidelines should be accessible to and consulted by physicians caring for PLWH. While HIV-treating physicians are familiar with guidelines from organizations such as the European AIDS Academic Society (EACS), World Health Organization (WHO), Centers for Disease Control and Prevention (CDC), and the German Standing Committee on Vaccination (STIKO—“Ständige Impfkommission”), these recommendations should also be known by general practitioners and physicians who encounter PLWH in their practice to recognize and address gaps in vaccination schedules.

Various vaccination methods differ both in their composition, such as live attenuated vaccines, inactivated vaccines, virus-like particles, polysaccharide vaccines, and mRNA vaccines, and in their routes of administration, including subcutaneous and intramuscular injections, as well as oral and nasal delivery. Of these vaccination types, live attenuated vaccines are those that require most caution in people living with HIV (PLWH), especially in individuals with CD4 cell counts < 200/μL, in whom the immune response is considered impaired. In these cases, live vaccines such as varicella, measles, mumps, rubella, and yellow fever, are contraindicated. Re-evaluation may be indicated once CD4 cell counts rise to >200/µL. Inactivated vaccines have fewer complications but may show differences in immunogenicity in comparison to the HIV-negative population. This leads to challenges in offering adequate protection to a vulnerable group and poses the question of whether sufficient efforts have been made in studying and developing vaccines for this more vulnerable group. Consequently, this highlights the need for research into both old and new improved vaccinations among the population of PLWH [5].

Our literature review focused on vaccines routinely recommended for HIV-infected individuals, focusing on their efficacy and safety, compared to the general population, to evaluate the depth of the scientific data available on this subject. We relied on evidence from studies and guidelines such as those from the WHO [6], CDC [7], EACS [8], and the STIKO [9].

We reviewed articles available on PubMed published between 2005 and 2025. Only articles written in English, German, and Spanish were considered for inclusion. We also included the German STIKO recommendations, EACS guideline recommendations, and CDC recommendations. The screening for suitability and record selection was performed by two individuals. The following terms were searched: (“HIV” OR “human immunodeficiency virus”) AND (“vaccination” OR “vaccine”) AND (“Hepatitis” OR “HPV” OR “Influenza” OR “Meningococcus” OR “Pneumococcus” OR “MMR” OR “Measles, Mumps, Rubella” OR “Varicella” OR “VZV” OR “Herpes” OR “Zoster” OR “Yellow Fever” OR “Travel vaccines” OR “SARS-CoV-2 Vaccination” OR “COVID 19 Vaccination” OR “MPX” OR “Monkeypox”).

The eligibility of included articles was assessed through a screening process that considered the title and abstract. Only the most relevant articles focusing on vaccines in HIV-positive adult patients were included for enhanced practical utility of the review article. Meta-analyses, randomized controlled trials, and trials with reported efficacy and safety outcomes were preferred. The references from the identified records were utilized to broaden the scope of the search. This systematic literature review was performed following the guidelines of the PRISMA Statement.

The conclusions presented in the tables are based on the authors’ findings from the cited studies. These results are further elaborated on and discussed in the Discussion section.

Available vaccines and their abbreviations were found and can be accessed in the Paul Ehrlich Institute (PEI) directory [10].

## 2. Relevant Sections

### 2.1. Standard Vaccines

Standard vaccines, also known as routine vaccines, are immunizations that are typically administered to individuals according to a predetermined schedule recommended for children, adolescents, and adults. This schedule is based on age, risk factors, and other considerations outlined by national and international health organizations such as the CDC and WHO, among others. These vaccines protect against common infectious diseases and are considered essential for public health [7,11]. Standard vaccines are recommended for the entire population, regardless of HIV status. While there may be slight variations from country to country, the basic standard remains consistent and aligns with WHO recommendation.

Vaccinations can be categorized into live attenuated and inactivated vaccines. Live attenuated vaccines are immunizations created using pathogens that have been weakened under laboratory conditions. These vaccines elicit a strong and long-lasting immune response by closely mimicking natural infections. Examples include the measles, mumps, and rubella (MMR) vaccine and the varicella vaccine.

Inactivated vaccines cannot cause infection, making them safer for immunocompromised individuals. However, they usually generate a weaker immune response compared to live attenuated vaccines, necessitating multiple doses and periodic boosters.

#### 2.1.1. Tetanus, Diphtheria, and Pertussis Vaccination

Tetanus, diphtheria, and pertussis vaccination (Tdap) is a part of routine vaccinations and can be administered to PLWH without restrictions. If the individual has not previously received Tdap at or after the age of 11 years, they should receive one dose of Tdap, followed by tetanus and diphtheria (Td) or Tdap every 10 years.

For those who have not received the primary vaccination series for tetanus, diphtheria, or pertussis, the recommended regimen is one dose of Tdap, followed by one dose of Td or Tdap at least 4 weeks later. A third dose of Td or Tdap should be administered 6–12 months after the initial dose (Tdap is preferred as the first dose and can substitute for any Td dose). Subsequently, Td or Tdap should be given every 10 years

Special precautions are necessary for individuals with open wounds. For those with three or more doses of tetanus toxoid-containing vaccine, Tdap or Td should be administered in the case of clean and minor wounds, if it has been more than 10 years since the last dose. For all other wounds, Tdap or Td should be administered if it has been more than 5 years since the last dose of tetanus toxoid-containing vaccine. Tdap is preferred for those without a prior Tdap or with an unknown history, and it is recommended for pregnant women needing a tetanus toxoid-containing vaccine [7,12].

Tetanus immune globulin (TIG) is recommended for individuals with wounds that are neither clean nor minor and who have received fewer than three prior doses of tetanus toxoid or have an unknown vaccination history. A single intramuscular dose of 500 units is generally recommended for both children and adults, with part of the dose infiltrated around the wound if identifiable. If TIG is unavailable, intravenous immune globulin (IVIG), which contains tetanus antitoxin, may be used as an alternative [13,14].

Vaccination against tetanus, diphtheria, and pertussis (Table 1) can be administered to PLWH without any restrictions. Studies have shown that PLWH exhibit a comparable rise in tetanus antibodies to that observed in HIV-negative control groups. However, there is a notable difference in the half-life of tetanus antibodies between populations, with a longer duration observed in the European population (37 years) compared to individuals of non-European origin (17.9 years). Although further research is required to elucidate the underlying causes of these differences, they suggest that different populations may require distinct vaccination schedules. This underscores the necessity for country-specific guidelines and the importance of monitoring antibody titers.

There is no evidence suggesting a faster decline in antibody titers based on CD4 cell count [15]. However, lower levels of diphtheria and pertussis antibodies have been observed in the HIV-infected group in comparison to the control group [17,19], particularly noticeable in the older population [17]. This finding highlights the need for increased monitoring and potentially more frequent booster vaccinations in older PLWH. In pregnant women, a decrease in pertussis antibody titers could pose a risk of inadequate protection for the infant [16], underscoring the significance of booster vaccines every 10 years and potentially indicating a need for more frequent booster vaccinations in different subpopulations of PLWH [15,19].

According to Troy et al., intradermal administration, although not a conventional method, allows for a 60% reduction in the standard dose of Inactivated Polio Vaccine [IPV] without compromising antibody titers. Despite this benefit, intradermal administration is associated with a higher incidence of adverse events compared to standard methods. Consequently, it is advisable to initiate vaccination according to established standard protocols before considering these alternative methods. Should the standard methods prove ineffective, newer methods may be explored [18].

#### 2.1.2. Hepatitis Vaccines

##### Hepatitis A Vaccine

Hepatitis A (HAV) vaccination is recommended in PLWH, especially in the presence of other risk factors, such as individuals traveling to regions with a high prevalence, individuals practicing anal sex or intravenous drug use (IVD), persons experiencing homelessness, those with an active hepatitis B (HBV) or hepatitis C (HCV) infection, or those with chronic liver disease. The vaccination schedule depends on the vaccine and consists of two-dose series of HAV at 0 and 6–12 months or a three-dose series of HAV-HBV at 0, 1, and 6 months. A booster shot is advisable after >25 years [7,12,20,21].

##### Hepatitis B Vaccine

The recommended schedule for Hepatitis B vaccination (HBV) includes either a monthly vaccination at month 0, 1, and 6 or a rapid immunization on days 0, 7, 21, and at 12 months. A booster vaccination can be considered every 10 years. Given the similar transmission routes of HBV and HIV, and the accelerated progression of liver disease in the presence of HIV, vaccination is generally recommended for all PLWH who are HBV-seronegative [7,12,20,21].

##### Hepatitis A and B

The suggested vaccination schedule for combined HBV and HAV vaccination includes doses at 0, 1, and 6 months [7,12,20,21].

The response to HBV and HAV vaccination is often suboptimal in individuals with HIV. Factors contributing to insufficient antibody levels include immunosuppression, indicated by low CD4 levels and high HIV RNA viral load, and the frequency and antigenic load in vaccine doses. The duration of the response is generally also poorer in PLWH (Table 2, Table 3 and Table 4).

Safety concerns are comparable to those in HIV-negative individuals. Measures to enhance vaccination response include starting ART before vaccination for those with CD4 counts < 200 cells/mm^3^, antibody testing post-vaccination, and re-vaccination if protective antibody levels are not yet achieved [31]. Annual serological testing is advised for low responders [35]. For HAV, routine vaccination is not widely implemented in PLWH but recommended for specific conditions (see above).

Other suggested strategies for managing and enhancing the response to HAV vaccination in HIV-infected adults include regular monitoring of antibody levels in vaccinated immunocompromised individuals, a booster dose every five years, and administering a three-dose schedule within a maximum of 12 months for individuals with a CD4 count below 300 cells/mm^3^ [21].

Seroconversion rates were similar for both three and two doses of the HAV vaccine in PLWH [22]. While there was a slight superiority in seroconversion rates with three doses, they were still lower compared to those seen in HIV-negative individuals [25]. A positive aspect was noted among PLWH with higher CD4 counts [24], indicating a sustained response to vaccination lasting up to 5 years [23]. However, an HCV coinfection was linked to a poorer serologic response [25]. Therefore, treatment for all cases of HCV should be administered and titers should be verified before proceeding with vaccination. It is advisable to adhere to standard protocols initially, prioritizing universal treatment and vaccination. Exceptions to this approach should only be considered after standard procedures have been followed and proven ineffective.

While the seroconversion rates regarding the HBV vaccine were linked to the CD4 cell count and viral load, these factors should not be grounds for delaying the vaccination of individuals at high risk, as antibody generation was observed across all CD4 counts and viral load levels [26]. However, vaccinating when the CD4 count is ≥200 cells/mm^3^ does ensure a better response [30,31]. Serological titers should undergo assessment both prior to vaccination and upon completion of the vaccination regimen. If seroconversion rates remain suboptimal, re-vaccination may be warranted once the CD4 count reaches ≥200 cells/mm^3^.

Various regimens have been proposed for PLWH, and both the regimen involving four intramuscular double doses [27] and the regimen with four intradermal low doses have demonstrated improved serological responses compared to the standard HBV vaccine regimen [28]. Suggestions have been made that individuals exhibiting an isolated anti-HBc profile, without an anti-HBs titer surpassing 100 mIU/mL four weeks after receiving a single recall dose of the Hepatitis B vaccine, should undergo supplementary vaccination with a reinforced triple double-dose regimen [29]. On the other hand, some sources suggest that increasing the dosage does not lead to higher response rates [31].

Individuals with HIV have demonstrated positive outcomes from adhering to vaccination and booster recommendations outlined in guidelines. This is particularly notable in the context of Hepatitis B, as evidenced by a study indicating a decrease in the prevalence of infection over time [36].

In individuals without prior HIV infection who did not respond to the standard hepatitis B vaccine, a notable efficacy was observed upon re-vaccination with a double dose of the combined HAV and HBV vaccine. This effectiveness could be attributed to the higher dosage, potential bystander effects from the Hepatitis A vaccine, or a combination of both [37]. Among PLWH, those with CD4 counts < 200 cells/mm^3^ and those with virologic suppression exhibited lower seroconversion rates to the HAV inactivated vaccine. Controversially, recipients of the inactivated HAV and recombinant HBV vaccine appeared to be more sensitive to these factors. Additionally, an incomplete vaccination was associated with a lower likelihood of response [34], highlighting the importance of adhering to controls and the vaccination schedules. In HBV vaccination low responders or non-responders, a new vaccination cycle can be considered; however, there are no standardized international indications. In the USA and France, a second series is recommended; however, in Britain, three vaccines with higher dosages are recommended.

The recently approved CpG-Adjuvanted HBV Vaccine demonstrates superior efficacy and achieves earlier seroprotection in comparison to current vaccines while maintaining a favorable safety profile. Its abbreviated two-dose regimen and elevated seroprotection rates, particularly within those with suboptimal vaccine response, render it a pivotal therapeutic option in HBV immunization [38,39].

#### 2.1.3. Human Papillomavirus Vaccine

The recommended Human papillomavirus (HPV) vaccination schedule involves doses administered at month 0, 1, and 6, with no need for a booster vaccine. Given the similarity in infection pathways with HIV and the heightened risk of cervical and anal cancer, especially in immunocompromised individuals, a full three-dose series is advised [7,12,20].

The STIKO guidelines [8] in Germany advise completing vaccination series for girls up to the age of 17, preferably before their initial sexual experience. Individuals 18 and older could benefit from an HPV vaccination but its effectiveness is reduced. As per the EACS, individuals undergoing HIV Pre-Exposure Prophylaxis (PrEP) should be provided with an HPV vaccine, along with HAV, HBV, and Mpox, in order to ensure enhanced protection against sexually transmissible infections. Additionally, vaccination is recommended between the ages of 9 and 45, with health insurance coverage varying based on age, gender, and sexual orientation. It is preferable to use the nonavalent vaccine. In individuals undergoing treatment for high-grade dysplasia, a complete vaccination course may be beneficial for secondary prevention [20].

The incidence of anal cancer is substantially elevated, ranging from 40 to 80 times higher, in PLWH compared to the general population [40]. Considering the heightened risk of cervical and anal cancer in individuals with HIV, it is advisable that patients undergo HPV vaccination and carry out regular screenings [41,42]. The shared link between HPV and HIV transmission is correlated with an elevated risk of transmitting one infection in conjunction with the other [43].

Four types of recombinant vaccines based on virus-like particles are currently available, including the bivalent, quadrivalent, and nonavalent vaccine (9vHPV) [44].

The majority of vaccinated individuals have shown a sustained immune response (Table 5) [45]. The vaccine is considered safe and well-tolerated [46,47], and early vaccination is recommended for enhanced protection against HSIL [45]. To date, there are not enough studies with estimates of the effects of HPV vaccines in PLWH. Efforts are being made to consider a single-dose vaccination in populations that cannot afford a three-dose scheme [48].

The efficacy of the 9vHPV vaccine, compared to the 4vHPV vaccine, demonstrates non-inferiority against HPV infection. Additionally, it shows superior efficacy against the five oncogenic HPV types exclusive to the 9vHPV vaccine. An Australian study suggests that, given the epidemiological landscape of HPV-associated cancers, the 9vHPV vaccine could potentially prevent an additional 15% of cervical cancers and up to 20% of other HPV-related cancers within the Australian population. Furthermore, there is evidence supporting a two-dose schedule with a 6–12 month interval between doses in young adolescents, as it yields comparable protection rates to those of a three-dose schedule [49]. Given this, it is worth considering whether catching up with the 9vHPV vaccine for individuals previously vaccinated with 2vHPV or 4vHPV is advisable.

Vaccination in advanced age due to heightened risk may be reimbursed by health insurance providers in Germany upon submission of an application. According to the German guideline on HPV vaccination, there is a strong consensus that HPV vaccination should be administered to PLWH in individuals up to the age of 26, following the vaccination schedule consisting of three doses [50]. In the USA, the HPV vaccine is recommended for all adults up to 26 years of age and for individuals between 27 and 45 years of age based on individual assessment.

#### 2.1.4. Influenza

The influenza vaccine is explicitly recommended for PLWH because of its association with elevated rates of pneumonia and an increased likelihood of severe invasive disease. Vaccination should be administered annually, and if possible, a 4-valent vaccine should be used [7,12,20].

The yearly occurrence of influenza outbreaks heightens the vulnerability of PLWH to experiencing severe complications. PLWH exhibited a faster fall in antibody titers compared to those without HIV infection (Table 6). Additionally, higher antibody levels were observed in individuals who had received a vaccination the previous year [51]. A study using a two-dose adjuvant influenza vaccine demonstrated a persistent immune response lasting up to one year [52]. Consequently, yearly booster vaccinations are essential for augmenting immunogenicity and ensuring heightened protection [53]. Among studies, the triple and inactivated influenza vaccine has been proven to be safe for PLWH [54]. Insufficient research has been conducted on the tetravalent vaccine, indicating a need for further studies.

As reported by Schwarze-Zander et al., a study examining the H1N1pdm09 vaccine in PLWH showed that prior to vaccination, 49% of individuals exhibited seroprotection. Following vaccination, the seroprotection rate increased to 66%. However, despite the initial high rates of seroprotection, only 7% of PLWH achieved seroconversion. Notably, no significant associations were found between seroprotection and seroconversion and current CD4+ T-cell count, HIV-RNA load, antiretroviral treatment, or demographic factors. Among PLWH with initially elevated H1N1 seroprotective titers, a single administration of the H1N1pdm09 AS03-adjuvanted vaccine elicited a modest antibody response.

These findings highlight the potential for the development of specialized vaccines tailored to unique circumstances to provide enhanced immunity and mitigate future outbreaks. Furthermore, they underscore the importance of sustained efforts in vaccine development and re-vaccination strategies to optimize immunity. Booster vaccinations are crucial for enhancing immunogenicity and ensuring heightened protection, although they remain challenging in PLWH [53].

#### 2.1.5. Meningococcal Vaccination

The tetravalent conjugate vaccine for Meningococcal serotype ACW135Y (MenACWY) is routinely recommended in individuals with HIV. In special situations, such as HIV infection, asplenia, persistent complement component deficiency, planning travel to countries with hyperendemic or epidemic meningococcal disease, or individuals in the military, re-vaccination is recommended every 5 years according to the CDC if the risk remains. Other guidelines, such as the German STIKO, do not support re-vaccination [7,12,20,56].

Meningococcus serotype B (MenB) vaccination is not part of the standard vaccination recommendations for PLWH. Instead, it is advised in accordance with national guidelines, focusing on the risk profile determined by factors like high-risk travel destinations, close contact with children, MSM, and specific conditions such as asplenia (including sickle cell disease), persistent complement component deficiency, or routine exposure to Neisseria meningitidis. The suggested number of doses ranges from 2 to 3, depending on age and the manufacturer’s guidelines [7,20].

The five primary serogroups for N. meningitidis are A, B, C, W, and Y. Currently, there are three types of vaccinations available: tetravalent (MenACWY), which covers serogroups A, C, W, and Y; monovalent vaccine against serogroup B (MenB); and monovalent glycoconjugate vaccine against serogroup C (Menjugate).

While there was no observed association between meningococcal vaccination response rates and CD4 cell count or viral load, PLWH exhibited comparatively lower response rates than HIV-naive patients (Table 7). Immunity was achieved within four weeks after vaccination with serogroups A, C, W-135, and Y. Previous vaccination correlated with higher immunogenicity after repeated vaccinations [57], reinforcing the established recommendation to re-vaccinate every 5 years in high-risk regions.

International guidelines propose diverse vaccination strategies against N. meningitidis. The EACS recommends administering the quadrivalent vaccine every 5 years based on risk factors and does not endorse the use of MenB vaccine. On the other hand, the National Institutes of Health (NIH) recommends vaccinating PLWH >18 years with the quadrivalent vaccine, while MenB vaccination is not routinely advised.

Studies indicate that meningococcal serogroup B vaccines, which are based on outer membrane vesicles, demonstrate promising cross-protection against gonorrhea.

Studies conducted in closed populations, such as those in Cuba and New Zealand [58,59,60], have explored the relationship between meningococcal vaccinations and gonorrhea incidence, revealing a promising correlation. For instance, the VA-MENGOC-BC vaccine was effectively utilized to control a meningococcal epidemic in Cuba. Following an increase in gonorrhea incidence, a substantial reduction in gonorrhea cases was observed subsequent to the widespread implementation of the VA-MENGOC-BC vaccination campaign [59]. Similar results have been seen in studies conducted in southern California, where the effectiveness of 4CMenB was studied [61]. The genetic homology between Neisseria meningitidis and Neisseria gonorrhoeae, which ranges from 80% to 90%, suggests a biologically plausible mechanism for cross-protection [62]. Efforts are underway to develop a more adapted vaccine based on these pathogen mechanisms to induce lasting immunity in at-risk populations, where antibiotic resistance is continually increasing [63].

#### 2.1.6. Pneumococcal Vaccination

Pneumococcal vaccination is expressly recommended in PLWH due to the increased likelihood of disease with a severe course.

According to the EACS, routine vaccination should take place in adherence to availability and national guidelines. Vaccination schemes should comprise one dose of a conjugated vaccine such as PCV-13, PCV-15, or PCV-20, even if previously vaccinated with the PPV-23 polysaccharide vaccine. Individuals vaccinated with PCV-13 or PCV-15 may be recommended to receive one dose of PPV-23 at least 2 months after the conjugate vaccine, according to some national guidelines [20].

In Germany, the latest national recommendations indicate that PLWH who are >18 years old are advised to receive PPV20, followed by a booster vaccination with PCV20 after 6 years [64]. For adults in groups who have already received PPSV23 or sequential vaccination with PCV13 or PCV15 followed by PPSV23, a PCV20 vaccination is recommended with a minimum interval of six years. For those aged 18 and older with risk factors for severe pneumococcal disease who have started sequential vaccination with PCV13 but have not completed it with PPSV23, PCV20 is recommended with a minimum interval of one year.

In general, PLWH demonstrated consistently lower protection rates against pneumococcal infection, and their immunity declined more rapidly compared to HIV-naive controls (Table 8) [65]. An improved immunological response was observed following immune system reconstitution. Further research is necessary to investigate newer agents such as adjuvanted vaccines, which could potentially offer enhanced protection for this patient population [66]. Since early 2022, the 20-valent pneumococcal conjugate vaccine (PCV20) has been approved in Germany for individuals aged 18 and older. After evaluating the available evidence on safety and efficacy and reviewing modeling results, the Standing Committee on Vaccination (STIKO) has concluded that PCV20 is superior to the previously recommended vaccines PPSV23 and PCV13. Currently, there are no data on the necessity of booster vaccinations following PCV20, so no recommendations can be made regarding this issue [67,68].

#### 2.1.7. Measles, Mumps, Rubella (MMR) Vaccine

PLWH with CD4 percentages ≥15% and a CD4 count ≥ 200 cells/mm^3^ for at least 6 months and lacking evidence of immunity to measles, mumps, or rubella are advised to undergo a two-dose series with a minimum interval of 4 weeks. As with all live vaccines, MMR vaccination is contraindicated in HIV infection with CD4 percentage <15% or CD4 count < 200 cells/mm^3^ and/or AIDS. There is a diminished protective effect after vaccination in the presence of unsuppressed viremia. In case of exposure and lack of prior vaccination, administration of immunoglobulins is recommended [7,12,20].

The initial humoral immune response to the MMR vaccine was similar between PLWH and HIV-uninfected controls (Table 9). Despite maintaining a high CD4 cell count and a sustained cellular proliferative response, individuals living with HIV experienced a rapid decline in antibodies within one year. No severe adverse effects were reported [69].

MMR antibody levels should be monitored, and if there is a decrease or absence of immunity, vaccination should be initiated.

#### 2.1.8. Varicella Vaccine

Serology testing should be conducted if there is a negative exposure history, and vaccination is indicated if the individual is seronegative. If serological values are adequate, vaccination is not necessary. The vaccination schedule entails a two-dose regimen administered at intervals of 2–3 months. Varicella vaccination is contraindicated for PLWH with a CD4 percentage <14% or CD4 count < 200 cells/mm^3^, applying the same CD4 threshold used for other live attenuated viral vaccines [7,12,20,70].

The Varicella (VAR) vaccine is recommended for PLWH due to the higher incidence and severity of both chickenpox and herpes zoster in this population. It can be implemented in PLWH with CD4 percentages ≥14% and CD4 count ≥ 200 cells/mm^3^.

#### 2.1.9. Varicella Zoster Vaccine

The varicella-zoster virus (VZV) vaccination is recommended for PLWH aged > 50. It comprises a two-dose series of recombinant zoster vaccine administered with an interval of 2–6 months (with a minimum interval of 4 weeks). For shingles prevention, it is advisable to prioritize the use of adjuvant recombinant sub-unit vaccines over live attenuated vaccines in accordance with national guidelines [7,12,20].

Herpes Zoster was common among PLWH before the introduction of ART and constitutes an indicator illness.

After the introduction of vaccines, the incidence of Herpes zoster could be reduced. Previous evidence regarding the Zoster (VZV) vaccine indicated that a three-dose subunit vaccine triggered an immune response and exhibited a clinically acceptable safety profile in PLWH (Table 10) [71].

More recent studies on Herpes Zoster rates revealed a higher incidence in PLWH, particularly those with low CD4 T-cell counts, especially when CD4 counts were <200 cells/μL and the viral load was unsuppressed. This trend was more prominent among PLWH under the age of 60 and, overall, when compared to HIV-uninfected individuals. The elevated rates of herpes zoster and the lower uptake of the zoster vaccine in PLWH have suggested the necessity for a safe and effective vaccine tailored specifically for this population, with potential extension to younger age groups [73]. Additional research is required to advance the development of an improved vaccine.

Regarding the live attenuated VZV vaccine, a two-dose regimen was deemed safe for PLWH with CD4 counts greater than or equal to 400 cells/μL. However, the serologic response rate was only modest for protecting PLWH against herpes-zoster [72].

According to the findings by Scherzer-Zander et al., a seronegative status for VZV was observed in 2% of the individuals studied. Additionally, in multivariable analysis, a younger age was notably correlated with seronegativity against all four viruses, namely measles, mumps, rubella, and VZV. These results highlight a significant necessity for MMR and VZV vaccination among individuals living with HIV in Germany who were born in 1970 or later. Consequently, the implementation of systematic screening for MMR and VZV antibodies, followed by vaccination, is strongly recommended among people living with HIV to mitigate the occurrence of serious diseases and complications associated with vaccine-preventable illnesses [74].

Considering the onset of herpes zoster at younger ages and the potential complications like encephalitis and meningitis, Zou et al. implies that adherence to ART may reduce the occurrence of VZV illness and associated hospitalization costs in PLWH. Furthermore, expanding the utilization of the shingles vaccine could also prove beneficial in this regard [75].

In specific cases, it is possible to submit a cost application to the health insurance company in Germany to request vaccination at a younger age. This option should be considered, especially since, as per Hawkins et al., the 50–59 age group among people living with HIV had the highest rates of herpes zoster [73].

#### 2.1.10. SARS-CoV-2 Vaccine

In a pandemic scenario, vaccination for all PLWH should align with national guidelines, regardless of their CD4 count and HIV viral load. For those with advanced HIV infection (CD4 count < 200 cells/μL) and detectable HIV viremia, a diminished humoral immune response is expected. In such cases, a COVID-19 booster dose is recommended. Bivalent COVID-19 vaccines are only approved for use in individuals with HIV who have already completed their primary vaccination against COVID-19 [20].

People with HIV face an elevated risk of contracting SARS-CoV-2 infection and experiencing severe COVID-19, especially when their CD4 cell count is low and their viral load is not suppressed [20]. In Germany, it is recommended to have received at least three vaccinations, preferably using mRNA vaccines, and annual booster vaccines can be considered [12]. According to German STIKO guidelines, a previous infection is considered a booster. Booster vaccinations are typically administered at intervals of ≥ 12 months from the last antigen exposure, with a preference for administration in the fall [76].

A robust humoral response was demonstrated in immunocompetent PLWH who received two doses of the SARS-CoV-2 vaccine (Table 11) [77]. Lower levels of anti-spike SARS-CoV-2 antibodies were observed in PLWH compared to HIV-negative individuals [78]. Re-vaccination schedules for PLWH are influenced by factors such as age, underlying conditions, and vaccine types, as well as immunity and the emergence of new variants [77]. The BNT162b2 mRNA vaccine appears to be safe and effective for PLWH [79].

In addition, it is imperative for clinicians to exercise caution when initiating COVID-19 treatment in patients with HIV, as careful consideration of potential drug–drug interactions and overlapping toxicities between COVID-19 treatments and antiretroviral medications is warranted [80,81].

Novel vaccines, particularly mRNA vaccines and adenovirus vector vaccines, have emerged as highly significant during the SARS-CoV-2 pandemic. Both types of vaccines have demonstrated favorable tolerability, scalability, and stability. Furthermore, the absence of vector immunity of mRNA positions them as strong candidates for future research, including applications targeting multiple pathogens. Adenovirus vector vaccines do not require a frozen cold chain, making vaccines more easily accessible in remote areas and low-income countries. Further research is required in this field, especially in PLWH [82,83].

#### 2.1.11. Respiratory Syncytial Virus (RSV) 

At present, there are no specific guidelines for the use of RSV vaccines in PLWH. The CDC and RKI recommends RSV vaccination for everyone aged 75 and older. Adults aged 60 to 74 who are at increased risk for severe RSV disease are also advised to receive the vaccine [84,85]. Further research is required to assess the effectiveness and the potential necessity of additional doses.

### 2.2. Travel Vaccines

Travel vaccines are immunizations recommended or required for individuals traveling to specific destinations where they may be at risk of contracting certain infectious diseases. The indication for travel vaccines is based on factors such as the traveler’s destination, the duration of their stay, their planned activities, and their current health status.

Vaccinating PLWH traveling to endemic regions, especially those at risk of experiencing a severe course of infection, such as newly diagnosed and/or treatment-naive PLWH, and particularly those with a CD4+ count of 200 cells/mm^3^ or lower, is crucial for preventing the cross-border spread of infections and minimizing the risk of severe illness and death. Most travel vaccines are considered safe for PLWH with a CD4+ count of 200 cells/mm^3^ or higher. To safeguard against preventable diseases, individuals should seek pre-travel consultations for recommendations on the necessary vaccines, including verifying their current vaccination status. If PLWH fall ill during their travels, seeking medical consultation is highly recommended [86].

A cautious approach to vaccination and travel is warranted for PLWH with compromised immune function, particularly those with a CD4 cell count below 200 cells/μL. Postponing vaccination and travel until immunological recovery is achieved, avoiding travel to endemic areas, seeking prompt medical attention if illness occurs during travel, and awaiting improved blood values before undertaking non-essential travel are essential measures to safeguard the health and well-being of immunocompromised individuals.

#### 2.2.1. Cholera (Oral Live Vaccine)

The cholera vaccination involves two doses administered with intervals ranging from over 1 week to less than 6 weeks. A booster vaccination is advised every two years [12]. It should only be given if strictly necessary.

The Oral Cholera Vaccine has demonstrated immunogenicity in PLWH, albeit with a suboptimal immune response. Vaccine immunogenicity appears to be influenced by factors such as viral load and CD4 count. Therefore, it should only be given when strictly necessary. Further investigation is warranted to gain a comprehensive understanding of these dynamics, which is essential for informing policy and guiding clinical practice effectively [87].

#### 2.2.2. Tick-Borne Encephalitis Vaccine

The tick-borne encephalitis (TBE) vaccine consists of two vaccinations spaced at intervals of 1–3 months, followed by a third vaccination after 5–12 months or rapid immunization. Booster vaccinations are recommended every 3–5 years [12].

In Germany, these vaccines are recommended in regions at risk of tick exposure, including occupational exposure, and are covered by German health insurance [76].

Jilich et al. found unsatisfactory immunogenicity after the rapid vaccination scheme, with early protection achieved in only a small proportion of recipients (Table 12). Overall limited knowledge exists regarding the mechanism of action of TBE vaccination in PLWH, suggesting further research is needed [88].

#### 2.2.3. Yellow Fever Vaccine

The yellow fever vaccination a live attenuated vaccine and is compulsory for travel to specific countries. It is administered as a single dose, with one booster vaccination recommended after 10 years. It is contraindicated in individuals with CD4 cell counts < 200/μL (14%) and/or AIDS, as well as in individuals with a history of/or current hematological neoplasia or thymus-related conditions (such as thymoma, resection, or radiation) [12,20].

Individuals with significant immune compromise, such as those with AIDS, are recommended to refrain from traveling to regions with a risk of yellow fever. In unavoidable circumstances, it is crucial to educate them on preventing mosquito bites and issue a medical waiver for vaccination [89].

The vaccine is based on a replicating a live attenuated virus. A single dose provides around 90% protection after 10 days and 99% protection after 30 days (Table 13). The decay pattern is similar in both PLWH and the general population. The yellow fever vaccine should be administered at least 10 days prior to travel, as this is the minimum period required for the development of immunity to the yellow fever virus [90].

Patients with perinatally acquired HIV exhibited a high seroconversion rate at levels exceeding the protective threshold following the initial yellow fever vaccination. However, a decline in antibody levels over time was observed, indicating that at least one booster vaccination may be necessary to maintain adequate circulating antibodies, contrary to the current recommendations for the general population [92], suggesting further research is needed.

#### 2.2.4. Japanese Encephalitis Vaccine

The Japanese encephalitis (JE) vaccine is recommended for individuals traveling to endemic areas for a duration of > 1 month or those at increased risk when the travel duration is unknown or <1 month. A two-dose series is advised before departure, administered on day 0 and day 28, with the last dose given at least 1 week prior to travel. Alternatively, a rapid immunization scheme on days 0 and 7 can be followed, preferably in conjunction with rabies vaccination. In the case of a repeated stay in an endemic area 12 months after basic immunization, a booster vaccination should be offered [12,93].

All travelers to JE-endemic countries should take precautions to avoid mosquito bites [93]. The WHO suggests that the JE vaccine can be administered to immunocompromised individuals, including PLWH, although the immune response may be lower compared to fully immunocompetent persons. Inactivated Vero cell-derived vaccines are recommended over live attenuated or live recombinant vaccines [94]. The immunogenicity and efficacy of JEV vaccination in PLWH inadequately studied. Among HIV-infected children, data suggests reduced antibody responses measured by geometric mean titers, but most still develop seroprotective responses [95].

As per the BHIVA Guidelines, no published studies have examined the safety, immunogenicity, and clinical efficacy of Japanese encephalitis virus (JEV) vaccination in HIV-positive adults. Consequently, there is inadequate evidence to support modifications in dosing or booster requirements compared to standard recommendations [96,97]. Further research is needed in PLWH.

#### 2.2.5. Rabies Vaccine

The rabies vaccination protocol is contingent upon the vaccination and immune status of the individual. In PLWH it is advisable to avoid rapid schedules [20].

For pre-exposure rabies vaccination in individuals with a CD4 count of ≥200 cells/μL, a two-dose intramuscular schedule is recommended on days 0 and 7. Should the individual present with a CD4 count of <200 cells/μL or detectable viremia, a pre-exposure vaccination with three doses on days 0, 7, 21, or 28 days should be considered, along with the measurement of the antibody titers 14 days later to assess the effectiveness of the vaccination.

In the case of rabies post-exposure prophylaxis (PEP) in unvaccinated individuals, immediate wound cleaning is crucial, and human rabies immunoglobulin (HRIG) should be infiltrated within and around the wound. Simultaneously, intramuscular administration of rabies vaccine should occur on days 0, 3, 7, and 14 if the CD4 cell count is ≥200 cells/μL. In the case of a CD4 cell count < 200 cells/μL or if detectable viremia is present, PEP should comprise a five-dose vaccination regimen on days 0, 3, 7, 14, and 28, accompanied by a dose of HRIG. If rabies serology demonstrates inadequate titers during the follow-up (antibody levels < 0.5 IU/mL), an additional vaccine dose is recommended.

In vaccinated PLWH, the PEP recommendation following a risky exposure involves a two/three-dose vaccination series on days 0 and 3 [20].

There exists a scarcity of data concerning the immunogenicity and clinical effectiveness of rabies vaccines utilized for pre- or post-exposure prophylaxis in PLWH. The available evidence suggests that vaccine immunogenicity is impacted by factors such as the CD4 cell count and viral load, often resulting in diminished or absent antibody responses. Caution is warranted when evaluating PLWH following potential rabies exposure, even in cases where immunocompromise is considered mild. Limited studies have reported that rabies vaccines were well tolerated in PLWH, even at double the standard dose [97]. Additional research is required to investigate the safety and immunogenicity of rabies vaccines in PLWH.

#### 2.2.6. Typhoid Fever Vaccine

Vaccination against typhoid fever is recommended for individuals traveling to endemic areas for more than 3–4 weeks [94]. The vaccination should be administered at least 2 weeks before potential exposure to ensure the adequate development of an immune response [86].

Available vaccines include both live vaccines, such as Ty21a administered orally, and inactivated vaccines, like Vi capsular polysaccharide (ViCPS), which is given as an injectable. The oral live attenuated typhoid vaccine is not recommended for immunocompromised individuals due to its live nature; hence, the inactivated ViCPS vaccine is preferred [94].

The oral live attenuated vaccine can only be administered to asymptomatic patients if their CD4 count is ≥200 cells/μL (≥15%), and it is contraindicated if the CD4 count is <200 cells/μL (<15%). In the latter case, the inactivated parenteral polysaccharide vaccine should be administered [20,94], especially in situations of significant typhoid exposure risk at the travel destination, despite its lower immunogenicity in immunocompromised individuals [86].

The oral live attenuated typhoid vaccination regimen consists of three doses on days 1, 3, and 5. A booster vaccination is recommended after 3 years for individuals who remain at risk [12]. The protection from the vaccine is effective for over 5 years [86].

The parenteral ViCPS typhoid vaccine is administered as a single intramuscular dose [12,89]. It provides protection for approximately 2 years [86]. A booster vaccination is recommended after 3 years [12].

In PLWH, the duration of protection from vaccines may be diminished, and the production of protective antibodies might be compromised, especially in those with CD4 cell counts below 200 cells/μL. However, there is currently no evidence supporting the need for adjustments in vaccine dosage or administration intervals [97].

#### 2.2.7. Monkeypox Vaccine

Indications for Monkeypox (Mpox) vaccination encompass risk factors such as being an MSM, being transgender or nonbinary, having received a new diagnosis of at least one sexually transmitted disease in the past 6 months, having multiple sex partners, engaging in sexual activities at commercial sex venues, participating in sexual encounters linked to large public events in areas with ongoing transmission of Mpox, being a sexual partner of individuals with the aforementioned characteristics, and anticipating involvement in any of the aforementioned situations [7].

PLWH with a CD4 count < 200 cells/μL (<15%) and/or detectable HIV viremia face an increased risk of potentially life-threatening opportunistic infection via Monkeypox (Mpox) infection [20,94].

Primary Mpox vaccination is recommended for PLWH, HIV pre-exposure prophylaxis (PrEP) users, and people at risk [20]. The dosing schedule comprises a two-dose series via subcutaneous administration on day 0 and 28 [7]. The intradermal route has also proven effective, using 1/5 of the standard dose [20].

In the event of a vaccine shortage, PLWH with CD4 T-cells < 350/uL or detectable HIV viremia should be given priority in vaccination and post-exposure prophylaxis (PEP) [7,20,94]. Data collected about PLWH with CD4 counts ≥ 100 cells/µL and ≤750 cells/µL show a lower immune response in HIV-infected individuals compared to non-infected individuals. No data are available on the immune response to MVA-BN (Mpox Vaccine) in other immunocompromised individuals [98].

After risky contacts, PEP with Mpox vaccination should be given as soon as possible, within 4 days of exposure. Administrating PEP 4 to 14 days after exposure may still offer some level of protection [20].

The emergence of mpox as an opportunistic infection underscores the need for ongoing HIV testing, prevention, and treatment to prevent HIV infection and disease progression, thereby reducing the risk and impact of severe mpox. Ensuring access to Mpox vaccination and sexual health services, including STI testing, for at-risk populations can further mitigate mpox as an HIV-associated opportunistic infection [99]. This indicates that the Mpox vaccine should be provided to all PLWH who are at risk of sexually transmitted infections.

Mpox vaccination demonstrated an increase in immune protection, rising from 35.8% after the first dose to 66% after the second dose, emphasizing the importance of administering two doses (Table 14) [100]. However, both natural and vaccine-induced immunity did not offer complete protection against Mpox infection. Nevertheless, the duration and severity of the disease were reduced [101]. Notably, viral blips, characterized by a short-term increase in viral load in individuals generally maintaining an undetectable viral load, were observed after Mpox vaccination. While these blips were rare, it is advisable to closely monitor the viral load in PLWH after vaccination [102]. Statistically significant evidence indicated that the smallpox vaccine, despite a reduced dose, remained effective against Mpox infection [103].

The Mpox live attenuated non-replicating modified vaccinia Ankara (MVA) strain vaccine is considered safe for individuals with HIV, although its effectiveness may be reduced if the CD4 count is <200 cells/μL or in those with unsuppressed HIV [8].

#### 2.2.8. Dengue Vaccine

The two licensed tetravalent live attenuated dengue vaccines, CYD-TDV and TAK-003, should not be administered if the CD4 cell count is below 200 cells/μL, consistent with the general contraindications for live vaccines [104]. For individuals with a history of previous dengue infection who are seropositive, the dengue vaccine is both safe and highly efficacious. However, in seronegative individuals, the vaccine has been associated with an increased risk of developing severe dengue approximately three years after vaccination if they contract a natural dengue infection. Consequently, the WHO recommends administering this vaccine exclusively to seropositive individuals and strongly encourages serological testing prior to vaccination to ensure appropriate candidate selection [105].

## 3. Conclusions

It is imperative for healthcare providers to regularly monitor the vaccination status and antibody levels in PLWH to ascertain which vaccinations need to be administered or updated.

Vaccination is especially important in immunocompromised individuals such as PLWH and therefore should adhere to the national guidelines intended for the general population. Inactivated vaccines usually present no complications; live vaccines (i.e., varicella, measles, mumps, rubella, and yellow fever), however, are not recommended in cases of severe immunodeficiency (CD4 cell count < 200/μL). Vaccination should preferably be administered after immune reconstitution and HIV viral load suppression to enhance immunogenicity. Repeated vaccinations may be necessary after immune reconstitution, and titers should be determined to check for immunogenicity. Vaccine responses in PLWH may be significantly lower due to reduced seroconversion rates and faster decline in antibody titers; therefore, rapid vaccination programs (e.g., Hepatitis A and B, tick-borne encephalitis, rabies, Japanese encephalitis) should be avoided due to the potential for a lower vaccination response. In cases of advanced immunodeficiency, it is recommended to assess and supplement the vaccination status of close contacts if needed. Regular vaccination counseling should be provided to PLWH, particularly prior to travel.

In some low- and middle-income countries, insufficient cold-chain infrastructure presents a major challenge for vaccine delivery. This is particularly relevant for PLWH, who may require timely and reliable access to vaccines to prevent severe infections. Further policy and planning are needed by governments to ensure that immunization plans align with infrastructure capacity. This could possibly be achieved by prioritizing certain vaccination groups, like PLWH, over others. Another possible solution could be integrated delivery systems that connect healthcare services, such as HIV clinics, with other hospitals to reduce logistical barriers. Mobile outreach services equipped with cooling equipment could also help extend the reach of vaccination programs. Overall, an important approach lies in vaccine development, by producing temperature-stable vaccines that can withstand storage at higher temperatures, which would reduce the dependence on refrigeration systems. By prioritizing thermostable vaccines for national immunization programs, coverage could be significantly improved [106,107].

The clinical efficacy of vaccines is often limited in PLWH, highlighting the need for further research to investigate vaccine tolerability, optimization of vaccination schedules, and the development of new vaccines specifically designed for immunocompromised individuals worldwide.

## Figures and Tables

**Table 1:** Tetanus, diphtheria, and pertussis vaccination response in PLWH compared to the general population.

Source	HIV Patients Included	Non-HIV Controls	Immune Response	Authors’ Conclusion
Dauby et al. 2022 [15]	103	-	- The documented date of tetanus toxoid immunization was obtained from medical records and a prospective study was conducted comparing demographic origin (Europe vs. non-Europe), viral load, CD4 cell count, and the antitetanus toxoid antibody (ATA) titers. - The research revealed a median of 0.926 IU/mL for ATA titers, and a range between 0.467 and 1.626 IU/mL was measured. Additionally, 6.8% of participants (7 out of 103) displayed ATA titers below 0.1 IU/mL. - The study estimated a half-life of 9.9 years for tetanus toxoid-specific antibodies within the entire cohort, with a 95% confidence interval ranging from 5.5 to 50 years. - There was no conclusive evidence indicating a difference in antibody decay rates based on CD4+ cell count. - Antibody titers are expected to dip below the WHO threshold of protection (0.1 IU/mL) after 37 years for the entire cohort and after 17.9 years for non-European individuals.	PLWH born outside Europe show a reduced half-life of tetanus toxoid-specific antibodies compared to the general population. This difference may be attributed to factors such as a lower nadir or current CD4+ T cell count and potential under-immunization in their country of origin before migration. Consequently, adhering to the longer intervals recommended for booster vaccinations in the general population may not be appropriate for this specific subgroup of PLWH.
Nunes et al. 2023 [16]	91	136	- One month after vaccination, pregnant women with HIV (PWWH) exhibited lower fold-increase and antibody concentrations for all epitopes compared to HIV-uninfected women. - A small proportion of PWWH achieved a ≥4-fold increase from pre- to post-vaccination for pertussis toxoid and pertactin or diphtheria IgG levels ≥ 0.1 IU/mL and ≥1 IU/mL post-vaccination. - Adverse events post-vaccination were similar in PWWH and HIV-uninfected individuals.	Tdap vaccination was both safe and immunogenic. PWHW exhibited diminished humoral immune responses, potentially impacting the vaccine’s efficacy in safeguarding their infants against pertussis when compared to infants born to HIV-negative women.
Boey et al. 2021 [17]	196	856	- Patients were included if they had received at least one pertussis-containing vaccine during adulthood or within the past 10 years. - Seroprotective titers for diphtheria, tetanus, and pertussis were observed in 29%, 83%, and 22% of participants, respectively. - Higher seroprotection rates were associated with vaccination within the last ten years.	Except for tetanus, a significant portion of at-risk individuals remains susceptible to vaccine-preventable diseases such as diphtheria and pertussis.
Troy et al. 2015 [18]	231	-	- Baseline immunity rates for poliovirus serotypes 1, 2, and 3 were 87%, 90%, and 66%, respectively. - After vaccination, there was a significant 64-fold increase in antibody titers. - The response to 40% of the standard intradermal dose was comparable to the standard intramuscular dose, resulting in slightly higher antibody titers. - Although intradermal administration had a higher incidence of local side effects, participants preferred it over intramuscular administration.	Intradermal administration (not a standard) enables a 60% reduction in the standard dose of Inactivated Polio Vaccine (IPV) without diminishing antibody titers.
Spina et al. 2018 [19]	30	30	- On day 28, PLWH showed a comparable increase in tetanus antibodies (geometric mean concentration, GMC, 15.6) to the control group (GMC, 23.1) but exhibited lower diphtheria antibodies [GMC, 2.3] than the control group (GMC, 16.4). The percentage of individuals who seroconverted for pertussis was lower in PLWH than in the control group (HIV, 62.1% versus control, 100%). Both groups developed a cellular immune response to tetanus, characterized by a Th2 (IL-4, IL-5, and IL-13) and Th1 (IFN-γ) response, with lower cytokine levels in PLWH compared to the control group. Specifically for pertussis, both cellular and humoral responses were less intense in HIV-positive adolescents, displaying a lower Th1 and Th17 profile and higher IL-10 levels.	Both groups exhibited good tolerance and developed an immune response following Tdap. Nevertheless, HIV-infected adolescents could benefit from more frequent booster doses.

**Table 2:** Hepatitis A vaccination response in PLWH compared to the general population.

Source	HIV Patients Included	Non-HIV Controls	Immune Response	Authors’ Conclusion
Tseng et al. 2013 [22]	140	217	- At the 48-week mark, seroconversion rates were 75.7%, 77.8%, and 88.5% for the two-dose regimen in HIV-infected, three-dose in PLWH, and two-dose in HIV-uninfected MSM (men who have sex with men) in the intention-to-treat analysis. - After three-doses, the GMT of anti-HAV antibody at week 48 was notably higher than that in two-dose scheme in HIV-infected MSM but lower than in HIV-uninfected MSM. - Multivariate analysis highlighted that higher CD4 counts and an undetectable plasma HIV RNA load before HAV vaccination were predictors of seroconversion in PLWH.	The serologic response rates for three and two doses of the HAV vaccine were comparable among PLWH. A higher CD4 count and effective suppression of HIV replication were associated with an increased seroconversion rate following HAV vaccination.
Jablonowska et al. 2014 [23]	234	-	- A favorable response (anti-HAV-T > 20 IU/L one month after the booster dose) was observed in 79.5% of patients. - In individuals with CD4 counts exceeding 200 cells/µL, multivariate analysis indicated that the presence of antibodies to the HCV was the sole prognostic factor influencing vaccination response. - Among responders available for follow-up, 82% (50 out of 61) maintained detectable anti-HAV-T levels at 1 year, and 75.5% (37 out of 49) at 5 years.	PLWH with elevated CD4 counts demonstrated a sustained response to vaccination, lasting up to 5 years. Individuals with both HIV and HCV coinfection exhibited a less favorable response to vaccination.
Kourkounti et al. 2013 [24]	113	-	- The immune response and GMC of anti-HAV were comparable between treated and treatment-naïve patients (78% vs. 76% and 237 mIU/mL vs. 158 mIU/mL). - Vaccine response was achieved in 71% of patients with a CD4 count of 200–499 cells/mm^3^, compared to 80% of participants with a CD4 count ≥ 500 cells/mm^3^ (*p* > 0.05). The critical factor influencing response was the CD4 T-cell count at the time of vaccination. Patients with a CD4 T-cell count ≥ 500 cells/mm^3^ were 4.3 times more likely to respond to the vaccine than those with a CD4 T-cell count of 200–499 cells/mm^3^.	The effectiveness of vaccination is linked to CD4 T-cell levels. The response to the HAV vaccine in PLWH with a baseline CD4 T-cell count exceeding 200 cells/mm^3^ is not predicted by the count of other immune cells or the use of antiretroviral therapy.
Launay et al. 2008 [25]	99	-	- By week 28, seroconversion, defined as having an anti-HAV antibody concentration > 20 mIU/mL, was observed in 82.6% and 69.4% of patients in the 3-dose and 2-dose groups, respectively. - Only 37.9% of patients demonstrated seroconversion after a single vaccine dose (intent-to-treat analysis). - The geometric mean titers of anti-HAV antibodies were 323 and 132 mIU/mL in the 3-dose group and 138 and 67 mIU/mL in the 2-dose group at 28 and 72 weeks following the initial vaccine dose.	The administration of three doses of the vaccine proved to be safe and resulted in elevated antibody titers.

**Table 3:** Hepatitis B vaccination response in PLWH compared to the general population.

Source	HIV Patients Included	Non-HIV Controls	Immune Response	Authors’ Conclusion
Bailey et al. 2008 [26]	503	-	- The immune response to the vaccine was linked to CD4 count and viral load at the initiation of vaccination. - Nevertheless, the production of HBV surface antibodies was observed across all CD4 counts and viral load levels. - In the multivariate analysis, only the HIV viral load was identified as a predictor of the immunologic response. - None of the vaccinated individuals contracted HBV.	HIV viral load outperformed CD4 count as a predictor of response to HBV vaccination. Nonetheless, neither a low CD4 count nor a high HIV viral load should serve as reason to postpone the vaccination of individuals at high risk.
Launay et al. 2016 [27]	437	-	- Patients were randomly assigned to one of three vaccination groups: the IM20 × 3 group (145 participants), receiving 3 intramuscular injections of the standard dose (20 μg) of recombinant HBV vaccine at weeks 0, 4, and 24; the IM40 × 4 group (148 participants), receiving 4 intramuscular injections of double doses (40 μg, 2 injections of 20 μg) at weeks 0, 4, 8, and 24; or the ID4 × 4 group (144 participants), receiving 4 intradermal injections of low doses (4 μg, one-fifth of 20 μg) at weeks 0, 4, 8, and 24. Follow-up visits were conducted at months 18, 30, and 42 to assess the duration of response and HBsAb titers. - At month 42, the response rates were 41% in the group receiving 3 intramuscular (i.m.) injections of the standard dose (20 μg HBV, IM20 × 3 group), 71% in the group receiving 4 i.m. injections of double doses (40 μg HBV, IM40 × 4 group), and 44% in the group receiving 4 intradermal injections of low doses (4 μg HBV, ID4 × 4 group). A notable percentage of patients, 15%, had HBsAb titers of less than 10 mIU/mL at 33.1 months in the IM40 × 4 group, 8.7 months in the IM20 × 3 group, and 6.8 months in the ID4 × 4 group.	IM40 × 4 regimen of the recombinant HBV vaccine enhanced the long-term immune response in comparison to the standard regimen.
Launay et al. 2011 [28]	437	-	- At week 28, the response rates were 65% in the IM20 × 3 group (n = 91), 82% in the IM40 × 4 group (n = 119), and 77% in the ID4 × 4 group (n = 108)	In PLWH, both the regimen involving four intramuscular double doses and the regimen with four intradermal low doses demonstrated enhanced serological responses compared to the standard HBV vaccine regimen.
Piroth et al. 2016 [29]	54	-	- At week 4, 46% of the patients (n = 25) exhibited a response. - In the multivariate analysis, only the ratio of CD4(+) T cells to CD8(+) T cells was associated with this response. - At week 28 and month 18, 58% of these patients (14 out of 24) and 50% (10 out of 20), respectively, maintained an anti-HBs level of ≥10 mIU/mL. - Among patients who did not respond at week 4 but received additional vaccinations, 89% (24 out of 27) and 81% (21 out of 26) had an anti-HBs level of ≥10 mIU/mL at week 28 and month 18, respectively. - The preS2-(activator protein)-specific interferon γ T-cell response increased between week 0 and week 28 in patients who eventually responded to reinforced vaccination.	Patients with an isolated anti-HBc profile, lacking an anti-HBs titer exceeding 100 mIU/mL four weeks after a single recall dose of HBV vaccine, should undergo additional vaccination with a reinforced triple double-dose regimen.
Fonseca et al. 2005 [30]	210	-	- The seroconversion rate (anti-HBs > or =10 mIU/mL) stood at 34% for the standard dose and 47% for the double dose. - A significantly higher seroconversion rate was noted with the double dose compared to the standard dose in patients with CD4 cell counts > or = 350 cells/mm^3^ (64.3% vs. 39.3%), while no notable difference was observed in those with CD4 <350 (23.8% vs. 26.3%). - The double dose demonstrated an improvement in seroconversion compared to the standard dose in patients with HIV viral load <10,000 copies/mL (58.3% vs. 37.3%), but there was no significant difference in those with HIV viral load > or =10,000 copies/mL (16% vs. 17%).	Administering a double dose as the primary series, especially when the viral load is anticipated to be low and CD4 counts are ≥350, ensures an optimal immune response.
Cornejo-Juárez et al. 2006 [31]	79		- A statistically significant higher seroconversion rate was observed in patients with CD4 cell counts at vaccination ≥ 200 cells/mm^3^ (86.8%) compared to those with CD4 <200 cells/mm^3^ (36.6%). - Logistic regression analysis confirmed that CD4 counts < 200 cells/mm^3^ were significantly associated with a non-serologic response. - No differences were found between the two vaccine doses.	Increasing the dose of the HBV vaccine did not lead to a higher response rate in HIV-infected subjects. The only significant factor associated with the response rate was having a CD4 count ≥ 200 cells/mm^3^. As a result, the study suggests using this threshold for initiating vaccination in HIV patients.
[32]	67	-	- A total of 37.3% of PLWH had failed at least two courses of CpG-adjuvanted vaccine vaccines. - A total of 86.6% of PLWH became seropositive (Anti-HBs > 10 mIU/mL) at least two months after completing the CpG-adjuvanted vaccine regimen. - Among the 13.4% who did not develop immunity, 33% had detectable HIV RNA, and 33% had a CD4 count < 200 cells/µL.	- CpG-adjuvanted vaccine proved to be effective in inducing immunity against HBV in PLWH who failed non-adjuvant recombinant vaccines.
[33]	64	-	- The overall seroprotection rate (SPR) was 81%, at 79% in individuals with no prior HBV vaccination and no anti-HBc positivity and 84% in those who had previously failed vaccination. - Lower CD4+ counts, both current and nadir, were associated with reduced seroprotection.	- CpG-adjuvanted vaccine should be considered for wider application in HBV vaccination for PLWH.

**Table 4:** Hepatitis A and B vaccination response in PLWH compared to the general population.

Source	HIV Patients Included	Non-HIV Controls	Immune Response	Authors’ Conclusion
Jimenez et al. 2013 [34]	226	-	- Patients who had received at least one dose of Havrix or Twinrix and had anti-HAV antibody data pre- and post-vaccination were included. - About 53.5% of the population studied experienced seroconversion. - Individuals who responded to the vaccination had higher baseline median CD4 counts (446 versus 362 cells/mm^3^; *p* = 0.004) and lower median HIV RNA levels (475 copies/mL versus 5615 copies/mL; *p* = 0.018) compared to non-responders. - Incomplete completion of the vaccination series was associated with a lower likelihood of response.	PLWH exhibit significantly lower seroconversion rates to HAV inactivated vaccine vaccination, with responses influenced by CD4 cell count and virologic suppression at the time of vaccination. Recipients of inactivated HAV and recombinant HBV vaccine seem more sensitive to these factors and to the completion of the vaccine series compared to those receiving solely inactivated HAV.

**Table 5:** HPV vaccination response in PLWH compared to the general population.

Source	HIV Patients Included	Non-HIV Controls	Immune Response	Authors’ Conclusion
Hidalgo-Tenorio et al. 2021 [45]	129	-	- A notable difference was observed in antibody levels against quadrivalent (qHPV) vaccine genotypes at 7 months (76.9% in the vaccine arm vs. 30.2% in the placebo arm), 12 months (68.1% vs. 26.5%), 24 months (75% vs. 32.5%), 36 months (90% vs. 24.4%), and 48 months (87.2% vs. 30%).	Nearly all vaccinated individuals exhibited a sustained immune response. The primary protective factor against ≥ HSIL was completing the vaccination regimen more than 6 months prior.
Boey et al. 2021 [46]	100	171	- Seroconversion for all HPV types occurred in all PLWH. GMTs ranged from 180 to 2985 mMU/mL in PLWH and from 17 to 170 mMU/mL in organ transplant recipients, depending on the specific HPV type. Injection site adverse events occurred in 62% of participants.	The nonavalent (9vHPV) vaccine demonstrates high immunogenicity in PLWH, although its efficacy is suboptimal in organ transplant recipients. Nevertheless, the vaccine is deemed safe and well-tolerated in both groups.

**Table 6:** Influenza vaccination response in PLWH compared to the general population.

Source	HIV Patients Included	Non-HIV Controls	Immune Response	Authors’ Conclusion
Mahdi et al. 2011 [54]	506	-	- In preventing confirmed influenza illness, trivalent, inactivated influenza vaccine (TIV) demonstrated an effectiveness of 75.5%, with a risk difference of 0.18 per 100 person-weeks among recipients. - Seroconversion, assessed by hemagglutinin antibody inhibition assay (HAI) titers, occurred in 52.6% for H1N1, 60.8% for H3N2, and 53.6% for influenza B virus in TIV recipients, while 2.2%, 2.2%, and 4.4% of placebo recipients experienced seroconversion.	In African HIV-infected adults without underlying co-morbidities, TIV vaccination has been found to be safe and effective. Further evaluation of its effectiveness is recommended, especially in severely immunocompromised HIV-infected adults and those with co-morbidities like tuberculosis.
Pariani et al. 2011 [51]	49	60	- Both HIV-infected and HIV-naive subjects met the EMA-CPMP criteria for immunogenicity of influenza vaccines, with no notable differences in vaccine responses between the two groups. - Six months post-vaccination, the percentages of individuals with antibody titers ≥1:40 and antibody GMT decreased significantly in both groups, with lower levels in PLWH compared to HIV-uninfected individuals. - Among those primed to seasonal influenza the previous year, antibody levels against 2009 A(H1N1) were higher than in unprimed subjects, both one month and six months post-vaccination.	The concurrent administration of a single dose of the 2009 pandemic MF59-adjuvanted influenza vaccine with a seasonal vaccine induced a protective immune response in both PLWH and HIV-naive individuals. Individuals previously primed to seasonal influenza in the preceding year exhibited a more robust and persistent antibody response to the 2009 pandemic vaccine.
Durier et al. 2013 [52]	309	-	- Two administrations of the AS03A-adjuvanted H1N1v vaccine, each containing 3.75 μg of haemagglutinin (n = 155; group A), or the nonadjuvanted H1N1v vaccine with 15 μg of haemagglutinin (n = 151; group B), were performed. - In both groups A and B, the seroprotection rates were 83.7% and 59.4% at month 6, and 70.4% and 49.3% at month 12, respectively.	In adults infected with HIV-1, two doses of adjuvanted influenza vaccine elicited a persistent immune response lasting up to 1 year post-vaccination.
Zhang et al. 2024 [55]	2436	-	- The pooled relative risk (RR) of seroprotection and seroconversion for two-dose vaccination (compared to a single-dose vaccination) was 1.14 (95% CI: 1.08–1.21) and 1.25 (95% CI: 1.16–1.34). - The likelihood of elevated body temperature in PLWH who received a two-dose vaccination was 3.42 times greater than those who received a single-dose vaccination. - Conversely, the probability of experiencing myalgia was 25% lower. - No severe vaccine-related adverse events were reported.	Administration of two vaccine doses resulted in better immunogenicity compared to a single dose. The safety profile was considered acceptable in PWLH. An adjuvant two-dose regime may offer advantages over the conventional schedule.

**Table 7:** Meningococcal vaccination response in PLWH compared to the general population.

Source	HIV Patients Included	Non-HIV Controls	Immune Response	Authors’ Conclusion
Siberry et al. 2010 [57]	319	-	- Baseline immunity to specific serogroups was associated with factors such as CDC stage B/C (for serogroups C, W-135, Y), older age (for serogroups C and Y), and nonperinatal HIV acquisition (for serogroups A, C, Y), while baseline CD4% and VL categories did not show any association. - Proportions of individuals exhibiting a ≥4-fold SBA titer rise to individual serogroups varied from 52% to 73%, with 88% showing a ≥4-fold rSBA titer rise to at least one serogroup. - When excluding subjects with baseline immunity, the proportions with ≥4-fold rSBA titer rise were similar for serogroups C, W-135, and Y (51%, 73%, and 65%, respectively) but higher for serogroup A (76%). - Higher baseline GMT for serogroups consistently correlated with higher 4-week GMT. - Serogroup C exhibited the lowest rates of response and immunity across all measures.	A considerable number of PLWH develop meningococcal immunity naturally. While MCV4 is deemed safe and immunogenic in PLWH, the response rates are comparatively lower than those observed in non-infected youth, especially among individuals with more advanced HIV clinical, immunologic, and virologic status.

**Table 8:** Pneumococcal vaccination response in PLWH compared to the general population.

Source	HIV Patients Included	Non- HIV Controls	Immune Response	Authors’ Conclusion
Garcia Garrido et al. 2022 [65]	80	40	- Two months after vaccination completion (Month 4), overall seroprotection was achieved by 49% of PLWH and 82% of controls. - Baseline seroprotection did not differ between the groups, but post-vaccination rates were consistently lower in PLWH for both PCV13+PPSV23 serotypes and those exclusive to PCV13 or PPSV23. - There were no differences based on baseline CD4 counts in PLWH, but those with a nadir CD4 count ≥ 200 cells/mm^3^ had higher overall seroprotection rates at 4 months and 12 months after enrollment. - Over 12 months, seroprotection increased significantly for all vaccine serotypes in both groups, with controls consistently having higher rates, except for specific serotypes. - In PLWH, a nadir CD4 count ≥ 200 cells/mm^3^ and co-administration of DTP were significantly associated with seroprotection at month 4.	While IgG levels increased significantly for all 24 vaccine serotypes in both PLWH and controls, only a minority of PLWH achieved seroprotection after PCV13 followed by PPSV23. Poor responders were associated with a CD4 nadir <200 cells/mm^3^. The DTP vaccine emerges as a potential enhancer of pneumococcal vaccination responses.
Slayter et al. 2013 [66]	79		- The odds ratios for delayed immunization, in comparison to immediate immunization, were 0.54, 0.341, and 0.204 at months one, six, and twelve, respectively. - Consistent distinction between immediate and delayed vaccination was evident across individual serotypes. - There was no significant distinction in the Opsonophagocytic activity (OPA) of antibodies responses (OPA scores > 2) between the two dosing strategies.	PLWHA displayed enhanced immunological responses to 23-valent pneumococcal polysaccharide (PPV) or pneumococcal heptavalent conjugate vaccine (7PCV) after immune system reconstitution. PLWHA should be vaccinated with pneumococcal vaccines when their CD4 count exceeds 200 cells/mm^3^. No superiority of recommending conjugate vaccines over polysaccharide vaccines for immunizing PLWH was established.

**Table 9:** Measles, mumps, and rubella vaccination response in PLWH compared to the general population.

Source	HIV Patients Included	Non-HIV Controls	Immune Response	Authors’ Conclusion
Belaunzarán-Zamudio et al. 2009 [69]	26	22-	- There was no significant difference in the humoral immune response to the vaccine between PLWH and the HIV-uninfected group at 3 months (81% vs. 86%, respectively). - One year after vaccination, a greater proportion of PLWH had lost measles antibodies compared to controls. - Despite the decrease in antibodies at 12 months, the cellular response showed no statistically significant differences between the groups at baseline, 3 months, and 12 months post-immunization. No severe adverse events were reported.	The initial immune response to the measles vaccine was comparable between PLWH adults and HIV-naive adults. PLWH exhibited a swift reduction in measles antibodies, despite maintaining a high CD4+ cell count and a sustained cellular proliferative response.

**Table 10:** Zoster and Varicella vaccine response in PLWH compared to the general population.

Source	HIV Patients Included	Non-HIV Controls	Immune Response	Authors’ Conclusion
Weinberg et al. 2010 [72]	67	15	- In PLWH vaccine recipients, VZV antigen-specific lymphocyte proliferation (RCF) significantly increased, and the Enzyme-Linked Immunospot (ELISPOT) assay showed a positive trend. However, there was no significant increase in any VZV CMI measures among PLWH who received the placebo. The vaccine’s immunogenicity did not demonstrate a correlation with the nadir CD4 cell counts in PLWH.	The administration of two doses of the live attenuated VZV vaccine was found to be safe in PLWH with CD4 counts greater than or equal to 400 cells/microL, though the immunogenic response was only modest.
Berkowitz et al. 2015 [71]	123	-	- Following the third dose of the Herpes Zoster Subunit (HZ/su) vaccine, recipients demonstrated elevated concentrations of serum anti-gE antibodies and increased frequencies of gE-specific CD4(+) T cells compared to the placebo group. - Peak cell-mediated immune responses occurred after the second dose, and both humoral and cell-mediated immune responses persisted until the study’s end at month 18.	HZ/su vaccine elicited an immune response and demonstrated a clinically acceptable safety profile in PLWH after 3 doses.

**Table 11:** SARS-CoV-2 vaccination response in PLWH compared to the general population.

Source	HIV Patients Included	Non- HIV Controls	Immune Response	Authors’ Conclusion
Brumme et al. 2022 [77]	100	152	- Considering multiple factors, HIV infection correlated with diminished anti-RBD antibody concentrations and ACE2 displacement activity following a single vaccine dose. - After receiving two doses, HIV did not exhibit a significant association with the magnitude of any humoral response when adjusting for various variables. Factors such as older age, a greater burden of chronic health conditions, and dual ChAdOx1 vaccination were identified as contributors to reduced responses after two vaccine doses. - There was no significant correlation found between nadir CD4+ T-cell counts and responses to two vaccine doses in PLWH.	PLWH who maintain suppressed viral loads and have CD4+ T-cell counts within a healthy range generate robust humoral responses following dual COVID-19 vaccination. Various factors, including age, co-morbidities, the vaccine brand, the longevity of the response, and the emergence of new SARS-CoV-2 variants, will play a role in determining when PLWH might derive additional benefits from supplementary doses.
Hensley et al. 2022 [78]	1154	440	- HIV positivity was linked to a diminished response. - All controls who received an mRNA vaccine demonstrated an adequate response, defined as >300 BAU/mL, whereas in PLWH, this response rate was 93.6%. - Among PLWH vaccinated with mRNA-based vaccines, a higher antibody response was anticipated in those with a CD4+ T-cell count of 250–500 cells/μL or >500 cells/μL, while a viral load > 50 copies/mL was associated with a reduced response	Following vaccination with BNT162b2 or mRNA-1273, PLWH exhibited lower anti-spike SARS-CoV-2 antibody levels compared to HIV-negative controls. Achieving and sustaining comparable serological responses to HIV-negative controls likely necessitates additional vaccinations.
Levy et al. 2021 [79]	143	261	- After the second dose, 98% of PLWH and 98.9% of healthcare workers (HCWs) developed positive anti-RBD-IgG at medians of 18 and 26 days, respectively. - After the second dose, immune sera effectively neutralized SARS-CoV-2 pseudovirus in 97% of PLWH and 98% of HCWs. - Adverse events, reported in 60% of PLWH, mainly included pain at the injection site, fatigue, and headache.	The BNT162b2 mRNA vaccine seems to be both immunogenic and safe for PLWH who are on antiretroviral therapy with an unsuppressed CD4 count and suppressed viral load.

**Table 12:** Tick-borne encephalitis vaccine response in PLWH compared to the general population.

Source	HIV Patients Included	Non-HIV Controls	Immune Response	Authors’ Conclusion
Jilich et al. 2021 [88]	28	-	- Seroprotection rates were 35.7% on day 28 and 39.3% on day 60. - There were no discernible differences in baseline and nadir CD4 + T lymphocyte levels between responders and non-responders. - Post-vaccination, no serious adverse effects were reported.	The study found unsatisfactory immunogenicity, with early protection achieved in only a small proportion of recipients of the rapid vaccination scheme, leading to the recommendation against rapid TBE vaccination for PLWH.

**Table 13:** Yellow fever vaccination response in PLWH compared to the general population.

Source	HIV Patients Included	Non-HIV Controls	Immune Response	Authors’ Conclusion
Motta et al. 2023 [91]	218	82	- The yellow fever (YF) vaccine was administered safely, with no serious adverse events reported. Seroconversion occurred in 98.6% of PLWH on day 30, and 100% of controls exhibited seroconversion. - After 1 year, 94.0% of PLWH and 98.4% of controls were seropositive. - PLWH showed lower GMTs than controls at both day 30 and 1 year. - Factors associated with lower YF neutralization titers included a baseline viral load >1000 copies/mL, a low CD4+ cell count, and a low CD4+/CD8+ ratio.	The YF vaccine is considered safe for PLWH with a CD4+ cell count ≥ 200 cells/μL. Immunogenicity of the YF vaccine is compromised in PLWH with high viral load, a low CD4+ cell count, and a low CD4+/CD8+ ratio at the time of vaccination. YF neutralization titers tend to decline over time in PLWH.

**Table 14:** Monkeypox vaccination response in PLWH compared to the general population.

Source	HIV Patients Included	Non-HIV Controls	Immune Response	Authors’ Conclusion
Deputy et al. 2023 [100]	2193	8319	- Adjusted vaccine effectiveness for two doses (full vaccination) in a group of 25 case patients and 335 control patients was estimated to be 66.0%. - For those who received one dose (partial vaccination), among 146 case patients and 1000 control patients, the estimated adjusted vaccine effectiveness was 35.8%.	Individuals with Mpox were less likely to have received one or two doses of the Mpox vaccine compared to control patients. The findings support the effectiveness of the Mpox vaccine in preventing Mpox disease, with a two-dose series providing improved protection.
Raccagni et al. 2023 [102]	187	-	- Six viral blips with an incidence rate of 1.59 per 100 person-months of follow-up (PMFU) and three confirmed viral failures (CVFs) with an incidence rate of 0.80 per 100 PMFU were observed. - Two CVFs occurred after the second vaccine dose, associated with detectable HIV-RNA following the first dose despite high compliance with antiretroviral therapy (ART). - Individuals experiencing viral blips or CVFs had a higher frequency of residual viremia before the first vaccination. - There were no significant differences in ART and the number of MBA-BN doses. - In two cases of CVFs, ART changes were made, and all viral blips resolved within a month.	While uncommon, instances of viral blips and CVFs were observed in PLWH receiving antiretroviral therapy (ART) following MVA-BN vaccination. It is advisable to closely monitor HIV-RNA levels during Mpox vaccination.
Martín-Iguacel et al. 2023 [103]	842	1280	- Smallpox vaccination was linked to a statistically significant reduction in the incidence of Mpox. - At the time of mpox diagnosis, 23.7% of PLWH had received at least one dose of the smallpox vaccine, with a median of 23 days between the first vaccine dose and symptom onset. A total of 16.5% of PLWH had received the smallpox vaccine in childhood.	Intradermal smallpox vaccination with reduced doses remained effective.
Hazra et al. 2024 [101]	8	29	- Individuals with natural immunity from the initial infection exhibited a shorter disease course and less mucosal involvement upon reinfection compared to their initial experience. - Post-vaccination infections were characterized by a few lesions, minimal mucosal disease, and lower analgesia requirements.	Neither natural nor vaccine-induced immunity provides complete protection against mpox infection. However, in this small series, both the duration and severity of the disease appear to be reduced.

## Data Availability

Not applicable.
